# Analysis of NOS Gene Polymorphisms in Relation to Cluster Headache and Predisposing Factors in Sweden

**DOI:** 10.3390/brainsci11010034

**Published:** 2020-12-31

**Authors:** Caroline Ran, Julia M. Michalska, Carmen Fourier, Christina Sjöstrand, Elisabet Waldenlind, Anna Steinberg, Andrea C. Belin

**Affiliations:** 1 Department of Neuroscience, Karolinska Institutet, 171 77 Stockholm, Sweden; juliammichalska@gmail.com (J.M.M.); carmen.fourier@ki.se (C.F.); andrea.carmine.belin@ki.se (A.C.B.); 2 Department of Clinical Neuroscience, Karolinska Institutet, 171 77 Stockholm, Sweden; christina.sjostrand@ki.se (C.S.); elisabet.waldenlind@sll.se (E.W.); anna.steinberg@sll.se (A.S.); 3 Department of Neurology, Karolinska University Hospital, 171 76 Stockholm, Sweden

**Keywords:** nitric oxide, nitric oxide synthase, neurovascular, smoking, haplotype

## Abstract

Cluster headache is characterized by activation of the autonomic-trigeminal reflex. Nitric oxide can trigger headaches in patients, and nitric oxide signaling is known to be affected in cluster headache. Based on the hypothesis of nitric oxide being involved in cluster headache pathophysiology we investigated nitric oxide synthases as potential candidate genes for cluster headache. We analyzed eight variants in the three forms of nitric oxide synthase (*NOS*) genes, inducible *NOS* (*iNOS*), endothelial *NOS* (*eNOS*) and neuronal *NOS* (*nNOS*), and tested for association with cluster headache. Swedish cluster headache patients (*n* = 542) and controls (*n* = 581) were genotyped using TaqMan^®^ assays on an Applied Biosystems 7500 qPCR cycler. This is the largest performed genetic study on *NOS* involvement in cluster headache so far. We found an association between cluster headache and one *iNOS* haplotype consisting of the minor alleles of rs2297518 and rs2779249 (*p* = 0.022). In addition, one of the analyzed *nNOS* variants, rs2682826, was associated with reported triptan use (*p* = 0.039). Our data suggest that genetic variants in *NOS* genes do not have a strong influence on cluster headache pathophysiology, but that certain combinations of genetic variants in *NOS* genes may influence the risk of developing the disorder or triptan use.

## 1. Introduction

Nitric oxide (NO) is a vasodilating molecule and neurotransmitter frequently discussed as a player involved in the pathophysiology of cluster headache (CH). NO is synthesized from L-arginine by means of several NO synthases (NOS). Three genes encode NOS enzymes, neuronal *NOS* (*nNOS* or *NOS1*), inducible *NOS* (*iNOS* or *NOS2*) and endothelial *NOS* (*eNOS* or *NOS3*), which differ by their expression patterns and regulation. *iNOS* expression is induced by cytokine stimuli in several cell types, namely, macrophages, hepatocytes and smooth muscle cells. *eNOS* and *nNOS* rely on the presence of Ca^2+^ and calmodulin, while *iNOS* is Ca^2+^-insensitive [1].

CH is classified as a trigeminal autonomic cephalalgia, the pathology characterized by excruciatingly painful attacks located around the eye. In active periods, these attacks occur at a frequency of one attack every other day to up to eight attacks per day. Active periods are usually followed by remission periods with no symptoms [2]. During CH attacks there is an activation of the autonomic-trigeminal reflex, where calcitonin gene-related peptide (CGRP) is released from trigeminal neurons innervating the dura mater, resulting in local inflammation and pain [3,4]. The mechanisms involved in triggering a CH attack remain unknown. NO is involved in many of the biological processes that drive CH attacks, e.g., vasodilation, inflammation and pain signaling [5,6], and signs of elevated levels of NO activity have been detected in patients [7]. Also, consistent with a role for NO in CH, NOS was shown to be localized to mast cells, nerve fibers and blood vessels in rodent dura mater [8,9]. Changes in NO levels or related molecules could therefore have an impact on CH pathophysiology.

The activation of the trigeminovascular system that occurs in CH is also observed in migraine and other primary headache disorders. Similarly, there are several lines of evidence that the involvement of NO could constitute a common pathophysiological feature for several forms of primary headaches. First, nitroglycerin, a compound known as an NO donor (e.g., the metabolization involves liberation of NO), is known to induce headache. When administered to primary headache sufferers (migraine, chronic tension-type headache or CH patients), nitroglycerin typically induces a headache with the same characteristics as those usually experienced by the patient, which suggests that NO is a common player in primary headaches [10,11,12]. Second, several studies investigated NO activity in connection to headache attacks and found signs of elevated NO activity in migraine patients. Increased NO metabolites were detected in the internal jugular blood, venous blood and platelets during a spontaneous migraine attack [13,14,15]. Furthermore, NO metabolites were suggested to be higher in patients during headache-free periods than in controls, but results are conflicting [16,17,18]. NO metabolites were additionally found to increase during nitroglycerin-provoked CH attacks [19]. CH patients have higher levels of NO metabolites in the cerebrospinal fluid in comparison to headache-free individuals. In addition, NO metabolites were also found to be slightly elevated in CH patients in remission [7]. This is of particular interest in light of the ground-breaking work of Dr. Ekbom, showing that nitroglycerin only triggers CH attacks in CH patients who are experiencing an active phase [12]. Together, these studies raise the possibility of a threshold level of NO which triggers a headache attack. Last, a few small candidate gene studies made attempts to investigate single nucleotide polymorphisms (SNPs) and short repeats in *NOS* genes as risk factors for migraine, so far with inconclusive results. Reports concerning polymorphisms in the *nNOS* gene showed no association with migraine [20,21,22]. A study on an *iNOS* polymorphism similarly lacked association, while a haplotype analysis identified an *iNOS* haplotype consisting of two markers (rs2779249-rs2297518), which was more common in migraine with aura [23,24]. One of the identified SNPs, rs2297518, further correlates with increased risk of migraine in interaction with another variant in *eNOS* (rs743506) [25]. Results from *eNOS* studies were also conflicting [26,27,28]. In concordance with *iNOS* results, additional associations with migraine with aura were discovered with *eNOS* haplotype analysis [29]. Genetic studies on *NOS* and CH are scarce. There is one previous report on a Swedish case-control material consisting of 91 CH cases and 111 controls, showing an association between a microsatellite marker in *iNOS* and CH [30].

As NO signaling and NOS enzymes are appealing candidates for CH, we screened eight markers in these genes and tested for association with CH. Two *iNOS* markers (rs2297518, rs2779249) and rs743506 in *eNOS* were included in our study as they were previously linked to migraine. The remaining five markers were selected from published data of associations with vascular disease, as well as SNPs having a possible effect on gene expression and function. We hypothesize that genetic variations in *NOS* could affect the activity of *NOS* genes and the production of NO and thereby contribute to the susceptibility of developing CH.

## 2. Materials and Methods

### 2.1. Study Material

The research described herein was performed in accordance with the Declaration of Helsinki. Study material was obtained after approval of the local ethics committee, the Swedish Ethical Review Authority in Stockholm, Sweden (diary number 2014/656-31/4), and receiving informed consent from Swedish CH patients and neurologically healthy controls living in Sweden. All patients were diagnosed with CH by a neurologist according to the International Classification of Headache Disorders (ICHD-III beta) criteria [2]. A total of 581 control individuals representing the general Swedish population and 542 CH patients were included (Table 1). These were part of a Swedish biobank for CH which was described previously [31]. Information about clinical aspects, medication and lifestyle was also obtained from the CH Biobank. The majority of the control individuals were anonymous blood donors; no information, except for sex, could be provided for these individuals.

### 2.2. DNA Isolation

DNA was purified from whole blood with the Puregene^®^ Blood Core Kit C (QIAGEN Sciences, Hilden, Germany). DNA concentrations were measured with the NanoDrop^®^ ND-1000 Spectrophotometer (NanoDrop Technologies Inc., Wilmington, NC, USA) and the isolated material was used for the screening of selected genetic variants.

### 2.3. Genotyping by Quantitative Real-Time Polymerase Chain Reaction

TaqMan^®^ quantitative Real-Time PCR (qPCR) (Applied Biosystems, Foster City, CA, USA) was used to determine the genotype of different SNPs in a Swedish CH case-control material, with the aim of characterizing susceptibility polymorphisms. Experiments were performed using an ABI 7500 FAST qPCR instrument (Applied Biosystems). PCR plates with 2–5 ng DNA were prepared and dried overnight. TaqMan^®^ SNP Genotyping Assays (Applied Biosystems) containing forward and reverse primers, as well as two 5′ fluorescently labeled (VIC (2-chloro-7phenyl-1,4-dichloro-6-carboxy-fluorescein), FAM (carboxyfluorescein)) allele-specific probes were 1:1 diluted with 1× TE (Tris EDTA) buffer. The following assays were used: C__15907244_10 (*nNOS*, rs2682826), C__86363451_10 (*nNOS*, rs41279104), C_1189257 (*iNOS*, rs2297518), C__3219460_20 (*eNOS*, rs1799983), C__15903863_10 (*eNOS*, rs2070744) and C__30245515_10 (*eNOS*, rs3918226). For two SNPs, we used custom-made assays previously published by others; 0184316890 (*iNOS*, rs2779249) and AHFBBRI (*eNOS*, rs743506) [24,32]. SNP assays were used with TaqMan^®^ Genotyping Master Mix (Applied Biosystems). The qPCR was performed using default fast-cycling conditions: pre-PCR read at 60 °C for 1 min, holding stage at 95 °C for 10 min, 50 cycles at 95 °C for 15 s and 60 °C for 1 min; post-PCR read at 60 °C for 1 min. The above cycling procedure was used for all but one SNP (rs2779249), for which the cycling stage was altered to 92 °C for 15 s and 60 °C for 1.5 min to increase the time for primer and probe binding, as well as for cleaving of the fluorescent probe by the polymerase. Allelic discrimination was performed during the post-PCR read with the 7500 Software v2.0.6 supplied with the instrument.

### 2.4. Statistical Analysis

Data was analyzed using PLINK v1.07 (http://pngu.mgh.harvard.edu/purcell/plink/) [33]. All SNPs were in Hardy–Weinberg equilibrium for both cases and controls. We used basic allele and genotype testing as well as logistic regression with sex as a covariate to evaluate genotype and allele associations and further verified our data with the Breslow-Day test for homogeneity. Multimarker analysis was performed in PLINK as a separate haplotype analysis for each gene. According to our power analysis (PS-Power and Sample Size Calculations v.3.1.2), we could detect odds ratios (OR) lower than 0.64 or higher than 1.5 with 80% power for SNPs with a minor allele frequency (MAF) of 20% in our sample size [34]. Stratified analysis for smoking, alcohol consumption and use of verapamil as a prophylactic treatment or triptans to abort attacks was performed in GraphPad Prism v.5.04 (GraphPad Softwares Inc, La Jolla, CA, USA).

## 3. Results

### 3.1. Single Marker Analysis

We genotyped eight *NOS* gene SNPs in 542 CH patients and 582 control individuals (Table 1) and analyzed these variants for association with increased risk of CH. We performed basic allele and genotypic tests to assess the association for each of the eight genetic variants. Genotypic analysis showed no association between these genetic variants and CH (data available upon request). Results from the allelic analysis are displayed in Table 2. Our data showed a slight overrepresentation of the minor allele of *iNOS* variant rs2779249 in CH patients. Statistic tests confirmed a weak association between this SNP and CH, which did not remain after correction for multiple testing. Further, allele frequencies in *eNOS* and *nNOS* variants as well as rs2297518 in *iNOS* were equally distributed in CH patients and controls.

As CH is more common in males than females (68% males in our material, Table 1), we verified our results using a logistic regression model with sex as a covariate, which showed no significance for these eight SNPs. In order to further verify that the trend for association with rs2779249 was not gender-specific, we ran a Breslow–Day test to analyze for heterogeneity between the male and the female population, but this test showed no variation between the two sexes (data available upon request).

### 3.2. Multimarker Analysis

Previous findings on *NOS* variants in relation to primary headache disorders showed interesting associations with haplotype analyses in addition to single variants. We therefore ran a haplotype analysis on each of the three *NOS* genes. We did not find any disease-associated haplotypes in *eNOS* and *nNOS*. Table 3 displays data from the *iNOS* gene. The haplotype consisting of the minor allele on both positions (AA), was slightly more common in the patient group, with a significant *p*-value of 0.022.

### 3.3. Subgroup Analysis Investigating Vasoactive Substances

As NO is known to act as a potent vasodilator, we further chose to perform a subgroup analysis stratifying for parameters known to affect blood vessel constriction. We used self-reported data obtained from questionnaires filled out by the patients at the time of their inclusion in our biobank [31]. The following parameters were selected for analysis: the use of triptans to abort an attack, use of verapamil as a prophylactic drug, alcohol as a trigger factor for attacks, and coffee and tobacco consumption. Coffee consumption was defined as low (no coffee or less than a cup per day), medium (one to three cups per day) or high (more than four cups per day). Tobacco use was defined as using or having used tobacco in the form of cigarettes or snuff compared to a group of patients having never used tobacco. Use of triptans and verapamil was self-reported at the time of data and material collection. Of the included patients, 51% reported that alcohol could trigger an attack, 31% used verapamil as prophylactic medication and 74% used triptans as acute abortive medication.

**Figure 1:**
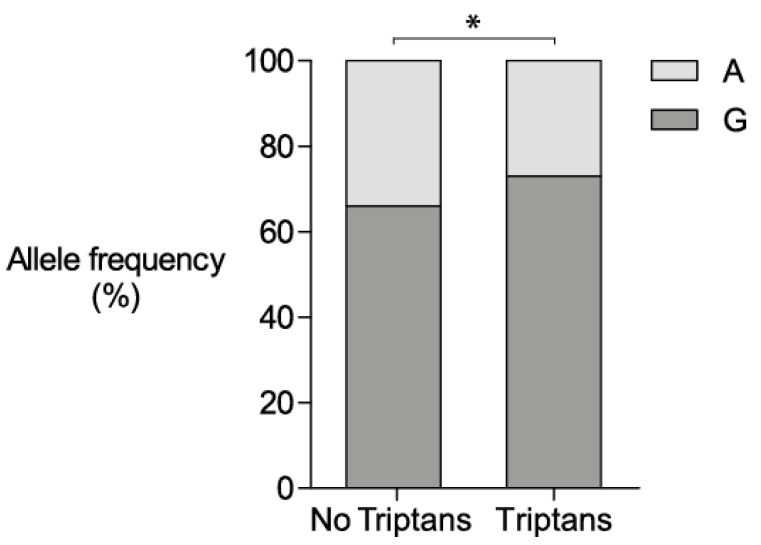
rs2682826 allele frequencies in patients using and not using triptans. Allele frequency of *nNOS* rs2682826 in triptan users (*n* = 388) vs. nontriptan users (*n* = 140). Analysis showed an association using Fisher’s exact test, two-tailed *p*-value, * = *p*-value < 0.05, OR = 0.72, 95% confidence interval = 0.55–0.98.

When comparing patients using and not using triptans we found an association with the *nNOS* rs2682826 SNP (Figure 1), *p*-value = 0.039, (OR 0.72 with a 95% confidence interval of 0.55–0.98). The minor allele (A) was overrepresented in the group of patients that did not use triptans (*n* = 140) with a minor allele frequency of 33.9%, compared to 27.3% in patients using triptans (*n* = 388), (for reference, 27.4% in controls). Coffee, tobacco and verapamil use as well as having alcohol as a trigger factor did not influence the distribution of alleles in patients (data available upon request).

## 4. Discussion

The aim of this study was to investigate genetic variants in *NOS* genes as potential risk factors for CH. We selected eight SNPs for analysis and one showed a trend for association with the disorder (rs2779249 in *iNOS*). Similar to other *NOS* candidate gene studies in headache, the strength of the association was insufficient, and significance was lost after correction for skewed gender distribution and multiple testing. The sample size in this study was over 500 cases and 500 controls, larger than most genetic studies published previously on *NOS* genes, but with an OR of 1.2, the discovered association falls outside the range of our 80% power detection limit (OR > 1.5 or <0.64). We performed a haplotype analysis on all three genes, which was in favor of association between *iNOS* and CH, but not *nNOS* and *eNOS*. The disease-associated haplotype was composed of two SNPs, both of which are biologically relevant, as they are expected to result in increased transcriptional activity of *iNOS* (https://gtexportal.org). Although our haplotype analysis supports the association between *iNOS* and CH, it is likely that the effect of these variants on the risk of developing CH is relatively small, a conclusion which was also suggested by Sjöstrand et al. in a previous study on *NOS* variants in Swedish CH patients [30]. Further analyses of these genes in independent cohorts are warranted in order to draw any final conclusions on the genetic contribution of *NOS* variants in CH. One reason for conflicting and inconclusive results in genetic studies of *NOS* in headache could be variability due to population background. This was demonstrated previously by Dong et al. in a meta-analysis reporting rs2070744 as a risk factor for migraine only in Caucasians [28]. Secondly, few studies analyzed identical SNPs, contributing to inconsistent results, specifically regarding haplotype analysis.

One potential limitation of our study is the nature of the control group. A majority of the control individuals are anonymous blood donors for whom we have limited information. The blood donors consists of healthy individuals resident in Sweden aged 18–60 years. Therefore, we cannot exclude the possibility that there could be cases of CH in the control group. However, due to the low incidence of CH, the probability is very small and the occasional occurrence as such should not significantly alter our statistical analysis. In addition, an average of 14% of our control group might also be considered to suffer from migraine, as this is the prevalence of migraine in the Swedish population. As some of the analyzed SNPs are also associated with migraine, these numbers are more likely to influence our analysis. Referring to allele frequencies in previously published studies, we found that our control population had lower MAFs for all three migraine-associated SNPs (rs2779249, rs2297518 and rs743506), which is why a direct comparison was not possible [24,25]. Nevertheless, as the minor alleles of rs2779249 and rs2297518 are associated with increased risk of migraine with aura and the observed relationship between controls of the different studies is inversed [24], we are confident in the validity of our results.

In a subgroup analysis, we identified an association between patients not using triptans and the minor allele of the *nNOS* SNP rs2682826. Our data do not allow speculations as to why 26% of our patients do not use triptans. This finding should therefore be interpreted with caution, although we hypothesize that not using triptans is because of lack of efficacy or because of severe side effects. To our knowledge, this is the first indication of a genetic correlation between *nNOS* and triptan response. rs2682826 is reported as an expression quantitative trait locus (eQTL) for the F-box protein 21 (*FBXO21*) gene. *FBXO21* is a subunit of an ubiquitin protein ligase involved in the innate immune system. Interestingly, a pharmacological compound (NXN-188) with dual activity as an nNOS inhibitor and serotonin 5-HT1B/1D receptor agonist was investigated as a new drug for migraine. The drug was shown to inhibit CGRP release from the dura mater and trigeminal ganglia, but the efficacy of the drug in migraine is still uncertain, and there are no studies on CH [35,36]. In an in vivo study, another nNOS inhibitor, NXN-323, was shown to counteract triptan-induced allodynia in rats. The authors further demonstrated that nNOS expression was increased in dural afferents originating in the trigeminal ganglia after sumatriptan treatment in these rats [37]. Similar events could be at play in the development of triptan-induced medication-overuse headache, suggesting a possible role for nNOS antagonists in future pharmacological treatment strategies for headache. The dynamic interplay of NO and CGRP in the trigeminovascular system was suggested to create an inflammatory loop involving neurons and glial cells, where CGRP released in the trigeminal ganglia increases the production of NO. Released NO further induces CGRP expression and facilitates its secretion by trigeminal neurons, thereby creating a feed-forward effect, leading to peripheral sensitization and arterial vessel dilation. It is therefore not unlikely that an optimal treatment effect could be achieved by targeting both pathways. Moreover, genetic predisposition to drug efficacy could well be determined by a combination of common small effect variants, such as rs2682826 in *nNOS*. With the development of personalized medicine, where treatment strategies can be adapted to the individual phenotype and genotype of each patient, knowledge of genetic variants with a potential effect on treatment efficacy are becoming increasingly important. As costs for genetic analysis are rapidly decreasing, evidence-based gene tests for drug screenings could be implemented in clinics in an effort to shorten the time from diagnosis to relief of symptoms for this vulnerable group of patients.

## 5. Conclusions

In conclusion, we performed the largest genetic analysis on *NOS* involvement in CH so far. Our results indicate that combinations of genetic variants in *iNOS* may influence the risk of developing the disorder. We also found an *nNOS* allele associating with the use of triptans, suggesting an impact on treatment response worth investigating further.

## Figures and Tables

**Table 1:** The Swedish case-control material.

	Cluster Headache	Controls
Number of individuals (*n*)	542	581
Male %	68.5	56.6
Chronic subtype %	10.1	NA
Average age (years)	52.3	NA
Age interval (years)	17–92	18–65
Age of onset (years)	31.5	NA

*n* = number, NA = not available/applicable.

**Table 2:** Results from the allele analysis for eight variants in endothelial nitric oxide synthase (*eNOS*), neuronal NOS (*nNOS*) and inducible NOS (*iNOS*).

Gene	SNP	Allele	Controls % (*n*)	Cluster Headache % (*n*)	χ^2^ *df* = 1	*p*-Value	*p*_c_-Value
*eNOS*	rs2070744	T	64.2 (723)	65.3 (701)	0.27	0.60	1
		C	35.8 (403)	34.7 (373)			
	rs3918226	C	92.0 (1043)	09.0 (997)	0	1	1
		T	8.0 (91)	8.0 (87)			
	rs1799983	G	72.1 (826)	71.5 (749)	1.03	0.1	0.8
		T	27.9 (320)	28.5 (299)			
	rs743506	A	80.5 (898)	77.5 (832)	2.97	0.085	0.68
		G	19.5 (218)	22.5 (242)			
*nNOS*	rs2682826	G	72.6 (825)	70.9 (749)	0.78	0.37	1
		A	27.4 (311)	29.1 (307)			
	rs41279104	C	86.7 (1007)	87.8 (938)	0.68	0.41	1
		T	13.3 (155)	12.2 (130)			
*iNOS*	rs2297518	G	82.7 (850)	80.0 (818)	2.37	0.12	0.96
		A	17.3 (178)	20.0 (204)			
	rs2779249	C	71.4 (800)	67.6 (731)	3.89	**0.049**	0.39
		A	28.6 (320)	32.4 (351)			

SNP = single nucleotide polymorphism, *n* = number of individuals included in analysis, χ^2^ *df* = 1 = chi-squared with one degree of freedom, *p*_c_-value = *p*-value corrected for eight tests.

**Table 3:** Results from the rs2297518|rs2779249 *iNOS* haplotype analysis.

Haplotype	Frequency in Controls	Frequency in CH	χ^2^	*p*-Value
GC	0.65	0.61	2.90	0.088
AC	0.071	0.066	0.17	0.68
GA	0.18	0.19	0.25	0.62
AA	0.10	0.13	5.21	**0.022**

GC haplotype = the common allele from both genetic markers, CH = cluster headache, χ^2^ = chi-squared with one degree of freedom.

## Data Availability

The data presented in this study are available on request from the corresponding author. The data are not publicly available due to privacy restrictions.
